# Supplementary material for: Design and optimization of soft finger actuators for rehabilitation applications: A combined finite element and neural network approach
Source: PLoS One. 2025 Oct 31;20(10):e0334011. doi: 10.1371/journal.pone.0334011 (PMC12578219; doi:10.1371/journal.pone.0334011)
Supplement: TPU material curve — This table presents the relationship between the load and the deformation that has been obtained from a tensile test for a standard Iso specimen test made of TPU. (PDF) [file pone.0334011.s001.pdf]

TPU Material Mechanical Properties		
**No.**	Load	Deformation
**1**	0.00038383	9.47126E-05
**2**	0.002461191	0.144745027
**3**	0.00459566	0.412745452
**4**	0.006733872	0.607007079
**5**	0.008865532	0.784631557
**6**	0.010996255	0.936864869
**7**	0.013125106	1.075733701
**8**	0.01525583	1.20710625
**9**	0.017393106	1.330143696
**10**	0.019521957	1.445395342
**11**	0.021648936	1.551570043
**12**	0.023780596	1.655938982
**13**	0.025916	1.747556452
**14**	0.028051404	1.841608268
**15**	0.030178383	1.92422949
**16**	0.032303489	2.006577476
**17**	0.034441702	2.082541233
**18**	0.036577106	2.15244284
**19**	0.03870783	2.22049055
**20**	0.040832936	2.285830679
**21**	0.042961787	2.345382601
**22**	0.045102809	2.402725986
**23**	0.047232596	2.46171374
**24**	0.049359574	2.512076874
**25**	0.051490298	2.565115736

**26**	0.053621957	2.607966306
**27**	0.055759234	2.655475331
**28**	0.057889957	2.692248177
**29**	0.060015064	2.734598995
**30**	0.062148596	2.770049551
**31**	0.064281191	2.810594606
**32**	0.066417532	2.842175975
**33**	0.068540766	2.876207971
**34**	0.070672426	2.905678488
**35**	0.072810638	2.939130035
**36**	0.074940426	2.968003115
**37**	0.077067404	2.992636087
**38**	0.0792	3.01610887
**39**	0.081330723	3.040661853
**40**	0.083469872	3.061957953
**41**	0.085594979	3.085575848
**42**	0.087721957	3.106855666
**43**	0.089859234	3.128587103
**44**	0.09199183	3.152366391
**45**	0.094124426	3.171696043
**46**	0.096251404	3.189977348
**47**	0.098380255	3.20904863
**48**	0.100518468	3.229910101
**49**	0.102648255	3.2485779
**50**	0.104780851	3.264683231
**51**	0.106906894	3.279160473
**52**	0.109035745	3.296120195
**53**	0.111176766	3.310096977

**54**	0.113305617	3.326588802
**55**	0.11543166	3.340856516
**56**	0.117563319	3.35154456
**57**	0.11969966	3.364844624
**58**	0.121834128	3.377258441
**59**	0.12396017	3.389752247
**60**	0.126085277	3.401005167
**61**	0.128226298	3.410177674
**62**	0.130357957	3.419721809
**63**	0.132486809	3.43240957
**64**	0.134617532	3.440002831
**65**	0.136744511	3.452609188
**66**	0.138885532	3.460169887
**67**	0.141013447	3.470068663
**68**	0.143142298	3.478338642
**69**	0.145272085	3.488527642
**70**	0.147404681	3.498377575
**71**	0.149541021	3.508807956
**72**	0.151668936	3.516030297
**73**	0.153796851	3.527831104
**74**	0.155931319	3.538165216
**75**	0.158063915	3.549401855
**76**	0.160197447	3.5587039
**77**	0.162323489	3.567038295
**78**	0.164454213	3.576598004
**79**	0.166592426	3.589543427
**80**	0.168723149	3.599167551
**81**	0.170850128	3.610259078

**82**	0.172981787	3.618062575
**83**	0.175112511	3.629040844
**84**	0.17725166	3.641696043
**85**	0.179378638	3.650224393
**86**	0.181506553	3.661509167
**87**	0.183641021	3.669263113
**88**	0.185770809	3.677533093
**89**	0.187907149	3.686674453
**90**	0.190034128	3.695605578
**91**	0.192158298	3.706196645
**92**	0.194300255	3.713532243
**93**	0.196431915	3.723431019
**94**	0.198561702	3.726977419
**95**	0.200687745	3.733458625
**96**	0.202819404	3.739229844
**97**	0.204957617	3.748692575
**98**	0.20708834	3.755754229
**99**	0.209211574	3.765475331
**100**	0.211346979	3.771858852
**101**	0.213481447	3.779049338
**102**	0.215615915	3.787625823
**103**	0.217742894	3.796782756
**104**	0.219865191	3.803714872
**105**	0.222009957	3.811017909
**106**	0.224138809	3.815048489
**107**	0.226271404	3.824366107
**108**	0.228400255	3.832587952
**109**	0.230526298	3.842245346

**110**	0.232668255	3.852175975
**111**	0.234795234	3.860607348
**112**	0.236923149	3.861348482
**113**	0.239054809	3.873262547
**114**	0.24118834	3.883741063
**115**	0.243323745	3.889770652
**116**	0.245452596	3.891979189
**117**	0.247574894	3.899733135
**118**	0.249715915	3.902861188
**119**	0.251845702	3.912485312
**120**	0.253979234	3.919643236
**121**	0.256107149	3.923641254
**122**	0.258234128	3.931137538
**123**	0.260375149	3.937538048
**124**	0.262504	3.943857153
**125**	0.264633787	3.95056346
**126**	0.266765447	3.95590005
**127**	0.268894298	3.961252212
**128**	0.271033447	3.969167551
**129**	0.273160426	3.973584625
**130**	0.275284596	3.980855808
**131**	0.277423745	3.985917746
**132**	0.279555404	3.99317194
**133**	0.281688	3.999024563
**134**	0.283814043	4.005907836
**135**	0.285940085	4.011921144
**136**	0.288084851	4.017386565
**137**	0.290214638	4.02349685

**138**	0.292338809	4.028897147
**139**	0.294471404	4.033668861
**140**	0.296604936	4.040584696
**141**	0.298739404	4.047146599
**142**	0.300871064	4.053272457
**143**	0.302991489	4.059737382
**144**	0.305132511	4.066524386
**145**	0.307262298	4.07368231
**146**	0.309394894	4.079163304
**147**	0.311524681	4.085757061
**148**	0.313649787	4.09402704
**149**	0.315791745	4.101168684
**150**	0.317920596	4.109503787
**151**	0.320051319	4.115984285
**152**	0.32218017	4.12219084
**153**	0.324309021	4.128994125
**154**	0.326449106	4.136249027
**155**	0.328577957	4.14268139
**156**	0.330704936	4.148178665
**157**	0.33283566	4.156303532
**158**	0.334971064	4.161978481
**159**	0.337107404	4.167733418
**160**	0.339234383	4.172973738
**161**	0.341356681	4.180244213
**162**	0.343496766	4.187160048
**163**	0.345592851	4.192802435
**164**	0.347761957	4.197557868
**165**	0.349888936	4.199766405

**166**	0.352018723	4.204506265
**167**	0.354156	4.210890493
**168**	0.356285787	4.215904297
**169**	0.358412766	4.220611595
**170**	0.360547234	4.224786579
**171**	0.362677021	4.230880583
**172**	0.364814298	4.234766051
**173**	0.366942213	4.24115028
**174**	0.369066383	4.247051037
**175**	0.371205532	4.253063637
**176**	0.373337191	4.259754371
**177**	0.375469787	4.26694415
**178**	0.377597702	4.274327883
**179**	0.379724681	4.279470517
**180**	0.381865745	4.28719261
**181**	0.383996426	4.292270829
**182**	0.386120596	4.30152474
**183**	0.388253191	4.306908756
**184**	0.39038766	4.316517307
**185**	0.392521191	4.319886742
**186**	0.394651915	4.326931408
**187**	0.396774213	4.333927939
**188**	0.398914298	4.341295392
**189**	0.401043149	4.348856091
**190**	0.403176681	4.354676152
**191**	0.405307404	4.361447583
**192**	0.407431574	4.368089474
**193**	0.40957166	4.372748637

**194**	0.411702383	4.37856799
**195**	0.41383217	4.385661499
**196**	0.415960085	4.393190345
**197**	0.418090809	4.399718978
**198**	0.420232766	4.405619735
**199**	0.422360681	4.41147165
**200**	0.424482979	4.416856374
**201**	0.426618298	4.424707298
**202**	0.428752766	4.43051037
**203**	0.430887234	4.434831882
**204**	0.433015319	4.440425426
**205**	0.435140426	4.447970553
**206**	0.437279574	4.454644298
**207**	0.439409362	4.459658102
**208**	0.441540851	4.464688186
**209**	0.443672766	4.469588731
**210**	0.445799574	4.476295746
**211**	0.447937872	4.481437673
**212**	0.45006766	4.486096836
**213**	0.452193617	4.491417144
**214**	0.454328085	4.496060027
**215**	0.456460851	4.503201671
**216**	0.45859617	4.51031146
**217**	0.460722128	4.513922276
**218**	0.462848085	4.520032562
**219**	0.464987234	4.525239612
**220**	0.467119149	4.532542649
**221**	0.469249787	4.540264741

**222**	0.471377447	4.546036667
**223**	0.473510213	4.55254902
**224**	0.475646383	4.558836979
**225**	0.477775319	4.563398457
**226**	0.479902553	4.56997664
**227**	0.482037021	4.577069441
**228**	0.484165532	4.583067176
**229**	0.486302979	4.58688752
**230**	0.488433617	4.592481772
**231**	0.490554894	4.597011397
**232**	0.492694043	4.603702131
**233**	0.49482766	4.609118709
**234**	0.496961277	4.616502442
**235**	0.499087234	4.62191902
**236**	0.50121234	4.6277228
**237**	0.503357021	4.63388122
**238**	0.505488085	4.639942663
**239**	0.507613191	4.644456714
**240**	0.509740851	4.651227437
**241**	0.511874468	4.658127699
**242**	0.514013617	4.664399377
**243**	0.516141702	4.670637078
**244**	0.518264681	4.675892971
**245**	0.52040383	4.682728817
**246**	0.522534468	4.690564168
**247**	0.524668085	4.698092306
**248**	0.526797021	4.70454095
**249**	0.52892383	4.712053515

**250**	0.531059574	4.718599136
**251**	0.533192766	4.72630424
**252**	0.535322553	4.732333829
**253**	0.53745234	4.73779925
**254**	0.539581277	4.744731365
**255**	0.541719574	4.752614143
**256**	0.543851064	4.758144687
**257**	0.54597617	4.764061018
**258**	0.548109787	4.771186381
**259**	0.550243404	4.777409216
**260**	0.55237617	4.784389467
**261**	0.55450383	4.788774687
**262**	0.556631915	4.793385007
**263**	0.558771064	4.798076025
**264**	0.5609	4.805218376
**265**	0.563030638	4.810489842
**266**	0.565160426	4.816277341
**267**	0.567290213	4.819823742
**268**	0.569429362	4.826803992
**269**	0.57156	4.831318751
**270**	0.573684255	4.837509025
**271**	0.575815745	4.843812558
**272**	0.577950213	4.849616338
**273**	0.580084681	4.854227366
**274**	0.582216596	4.858660721
**275**	0.584337872	4.865301904
**276**	0.586477872	4.873056558
**277**	0.588610638	4.879069866

**278**	0.590741277	4.88550223
**279**	0.592867234	4.891434841
**280**	0.594994043	4.896014016
**281**	0.59714	4.902494514
**282**	0.59926766	4.908394564
**283**	0.601391915	4.916068521
**284**	0.603524681	4.923226446
**285**	0.605657021	4.92906208
**286**	0.607793617	4.937332059
**287**	0.609923404	4.943990939
**288**	0.612049362	4.950406314
**289**	0.61418383	4.956839386
**290**	0.616316596	4.96373894
**291**	0.618448936	4.970671055
**292**	0.620579574	4.979054293
**293**	0.622704681	4.984986905
**294**	0.624841277	4.992386211
**295**	0.626975745	4.998738586
**296**	0.629104681	5.007508317
**297**	0.631234468	5.015197848
**298**	0.633364255	5.023259008
**299**	0.635501277	5.030496921
**300**	0.637633191	5.036559071
**301**	0.63976383	5.04303957
**302**	0.641892766	5.05011609
**303**	0.644025106	5.056113825
**304**	0.646157872	5.063497558
**305**	0.648285532	5.06980109

**306**	0.650411915	5.075298365
**307**	0.652554468	5.082520705
**308**	0.654684255	5.086728251
**309**	0.656813191	5.09227366
**310**	0.658940426	5.097819778
**311**	0.661073617	5.103832378
**312**	0.663223404	5.110216606
**313**	0.665344681	5.116762228
**314**	0.667464255	5.123259008
**315**	0.669599574	5.12780562
**316**	0.671734043	5.132819424
**317**	0.673868511	5.140751044
**318**	0.675995319	5.144700219
**319**	0.678119574	5.150439584
**320**	0.680259574	5.157000779
**321**	0.682393191	5.163658951
**322**	0.684521277	5.169124372
**323**	0.686651064	5.17631415
**324**	0.688778723	5.184310186
**325**	0.69092	5.19287039
**326**	0.69304766	5.200883415
**327**	0.695175745	5.206816026
**328**	0.697306383	5.21466695
**329**	0.699439149	5.215860409
**330**	0.701575319	5.201012246
**331**	0.703705957	5.197465138
**332**	0.705833191	5.212602817
**333**	0.707962979	5.222614851

**334**	0.710098298	5.231239471
**335**	0.712233617	5.239010406
**336**	0.714360426	5.246490408
**337**	0.716488511	5.253454378
**338**	0.718624681	5.263368727
**339**	0.720759149	5.270382247
**340**	0.722886383	5.279844978
**341**	0.725015319	5.285826432
**342**	0.72714766	5.293661075
**343**	0.729282128	5.300271112
**344**	0.731414043	5.307686699
**345**	0.733537021	5.315699016
**346**	0.73567617	5.3249685
**347**	0.737808085	5.332642458
**348**	0.739938723	5.342170312
**349**	0.742066383	5.349473349
**350**	0.744195319	5.357921002
**351**	0.746333617	5.367319318
**352**	0.748468936	5.37554187
**353**	0.750593191	5.383037446
**354**	0.752722128	5.388921923
**355**	0.754856596	5.395902173
**356**	0.756991064	5.403640546
**357**	0.759124681	5.410943583
**358**	0.761246809	5.418536844
**359**	0.763381277	5.426742408
**360**	0.765517447	5.434497062
**361**	0.767648511	5.442122177

**362**	0.769778298	5.449021731
**363**	0.771901277	5.456518723
**364**	0.774041277	5.461773908
**365**	0.776173191	5.468254406
**366**	0.77830383	5.473654704
**367**	0.780432766	5.479894528
**368**	0.782561702	5.486278049
**369**	0.784698723	5.493259008
**370**	0.786831489	5.498417923
**371**	0.788957447	5.504286119
**372**	0.791088085	5.511315212
**373**	0.793220851	5.517311531
**374**	0.79536	5.522857648
**375**	0.797489787	5.529370709
**376**	0.799614043	5.534497062
**377**	0.801744681	5.541122673
**378**	0.803882979	5.546701352
**379**	0.806015319	5.553922984
**380**	0.808141277	5.561452538
**381**	0.810269362	5.567287464
**382**	0.812408511	5.577654137
**383**	0.814542128	5.58381185
**384**	0.816665106	5.592968783
**385**	0.818797872	5.598676294
**386**	0.820930638	5.607172082
**387**	0.82306383	5.614426984
**388**	0.825193617	5.623873434
**389**	0.827284255	5.631015078

**390**	0.829455319	5.64146174
**391**	0.831591489	5.648427125
**392**	0.833719574	5.658405182
**393**	0.835849362	5.664837545
**394**	0.837978298	5.672834289
**395**	0.840114468	5.680862179
**396**	0.842250638	5.690341898
**397**	0.844375745	5.698934664
**398**	0.846504681	5.708398103
**399**	0.848637447	5.714620939
**400**	0.850771915	5.725132017
**401**	0.852906383	5.732031571
**402**	0.855028511	5.741414313
**403**	0.857161277	5.748104339
**404**	0.859300426	5.756342465
**405**	0.861431064	5.763177603
**406**	0.86356	5.772028739
**407**	0.865682128	5.777171374
**408**	0.867822128	5.785134848
**409**	0.869957872	5.790777943
**410**	0.872085532	5.799240462
**411**	0.874214468	5.804787287
**412**	0.876347234	5.812831457
**413**	0.878483404	5.819376371
**414**	0.88061617	5.82529341
**415**	0.882741277	5.832805974
**416**	0.884868936	5.841205493
**417**	0.887007234	5.848443406

**418**	0.88914	5.856810363
**419**	0.891271489	5.863919445
**420**	0.893396596	5.872593615
**421**	0.895528085	5.879251079
**422**	0.897666383	5.887053868
**423**	0.899794468	5.896484746
**424**	0.901924255	5.903804063
**425**	0.904017447	5.912912862
**426**	0.90618766	5.920505415
**427**	0.90832383	5.929324697
**428**	0.910447234	5.936739577
**429**	0.912578723	5.945945353
**430**	0.914714043	5.951313088
**431**	0.916845957	5.960357472
**432**	0.918975745	5.965983578
**433**	0.921104255	5.973641254
**434**	0.923237021	5.980073618
**435**	0.925375319	5.987312239
**436**	0.927502128	5.996791251
**437**	0.929632766	6.002772705
**438**	0.931761702	6.009027394
**439**	0.93389617	6.017362497
**440**	0.936029787	6.024245771
**441**	0.938157872	6.03017909
**442**	0.940284681	6.038078148
**443**	0.942420851	6.045058399
**444**	0.944554468	6.052700503
**445**	0.946688085	6.060148652

**446**	0.948809362	6.067999575
**447**	0.950941277	6.07577051
**448**	0.953081277	6.084765343
**449**	0.955212766	6.093116019
**450**	0.957340851	6.103933602
**451**	0.95946766	6.11302612
**452**	0.961605106	6.122650244
**453**	0.963741277	6.131114179
**454**	0.965866383	6.140061584
**455**	0.967993617	6.14983082
**456**	0.970129787	6.15860126
**457**	0.972265106	6.16935372
**458**	0.974397021	6.176382105
**459**	0.976522128	6.185056275
**460**	0.978649787	6.193954838
**461**	0.980788936	6.202869682
**462**	0.982921702	6.212429391
**463**	0.985050638	6.220409853
**464**	0.987176596	6.229180293
**465**	0.989311915	6.238223968
**466**	0.991446383	6.24675161
**467**	0.993575319	6.255779713
**468**	0.995705106	6.261165145
**469**	0.997838723	6.269547675
**470**	0.999969362	6.27859135
**471**	1.00210383	6.286652509
**472**	1.004230638	6.295148298
**473**	1.006361277	6.302709705

**474**	1.008498723	6.311092235
**475**	1.01062766	6.318620372
**476**	1.01276	6.325875982
**477**	1.014886383	6.332710413
**478**	1.017017872	6.340110427
**479**	1.01915617	6.345527005
**480**	1.021285106	6.35339421
**481**	1.023412766	6.361373965
**482**	1.025540851	6.371821335
**483**	1.027680851	6.380075034
**484**	1.029813617	6.388974305
**485**	1.031938723	6.396293622
**486**	1.034061702	6.404853826
**487**	1.036205532	6.41260848
**488**	1.038338298	6.423989524
**489**	1.040469787	6.432308346
**490**	1.042591064	6.442207121
**491**	1.044728511	6.450508954
**492**	1.046864681	6.460988179
**493**	1.048994468	6.469806753
**494**	1.051123404	6.48207475
**495**	1.05325234	6.489861259
**496**	1.05538766	6.50017909
**497**	1.057522128	6.51023855
**498**	1.059649362	6.519024563
**499**	1.061775319	6.528939619
**500**	1.063910638	6.538579316
**501**	1.066045957	6.548977844

**502**	1.068177872	6.557877115
**503**	1.070305532	6.566018263
**504**	1.072431489	6.575594252
**505**	1.074571915	6.584734905
**506**	1.076703404	6.593489063
**507**	1.078831489	6.600582572
**508**	1.080958298	6.608723013
**509**	1.083094468	6.616913003
**510**	1.085228085	6.623765131
**511**	1.087358723	6.632229065
**512**	1.089485106	6.639983011
**513**	1.091621277	6.647625115
**514**	1.093751915	6.655539747
**515**	1.09588766	6.662375593
**516**	1.098013617	6.670129539
**517**	1.100143404	6.678286968
**518**	1.102278723	6.68766971
**519**	1.104410213	6.696197353
**520**	1.106542128	6.706031712
**521**	1.108668085	6.71465704
**522**	1.110800851	6.724378141
**523**	1.112937872	6.733196716
**524**	1.115065957	6.743868479
**525**	1.117191064	6.752864019
**526**	1.119327234	6.763746018
**527**	1.121462553	6.774788702
**528**	1.123597021	6.784139591
**529**	1.125718298	6.792909323

**530**	1.127848085	6.803097614
**531**	1.129988511	6.812689885
**532**	1.132118298	6.82249239
**533**	1.134250638	6.830181921
**534**	1.136376596	6.838597013
**535**	1.13851234	6.848543923
**536**	1.140646809	6.856765768
**537**	1.142774468	6.865503646
**538**	1.144903404	6.874144546
**539**	1.147034894	6.882624053
**540**	1.149166809	6.891361931
**541**	1.15130383	6.901131875
**542**	1.153431064	6.909789056
**543**	1.155557021	6.920783606
**544**	1.15769234	6.929328237
**545**	1.15982766	6.938484462
**546**	1.161960426	6.946545622
**547**	1.164085532	6.956379274
**548**	1.166215319	6.966712678
**549**	1.168353617	6.977175621
**550**	1.170485957	6.98855808
**551**	1.17261234	6.997037588
**552**	1.174740851	7.006854959
**553**	1.176876596	7.01954272
**554**	1.179011915	7.030504707
**555**	1.181140851	7.040194663
**556**	1.183268511	7.050431089
**557**	1.185401277	7.059410349

**558**	1.187531064	7.07093792
**559**	1.189670213	7.080965527
**560**	1.191794468	7.09170383
**561**	1.193925957	7.101727189
**562**	1.196061277	7.112479649
**563**	1.198193191	7.121688964
**564**	1.200320851	7.132646705
**565**	1.202450638	7.139661641
**566**	1.204582553	7.150138034
**567**	1.206721702	7.158894316
**568**	1.20884766	7.169972393
**569**	1.210973617	7.178565867
**570**	1.213111915	7.190012034
**571**	1.215206809	7.198152474
**572**	1.217377872	7.210065831
**573**	1.219502979	7.218482339
**574**	1.221631915	7.227911092
**575**	1.223771064	7.236214341
**576**	1.225899149	7.244482197
**577**	1.228035319	7.251412189
**578**	1.230157447	7.261315212
**579**	1.232288511	7.268535429
**580**	1.234428511	7.279627663
**581**	1.236559149	7.287782261
**582**	1.238684255	7.297246408
**583**	1.240815745	7.30574078
**584**	1.24295234	7.3168047
**585**	1.245085957	7.324300984

**586**	1.247211915	7.333828838
**587**	1.249337872	7.342563885
**588**	1.251477021	7.352254548
**589**	1.25361234	7.362730941
**590**	1.255741277	7.373292277
**591**	1.257869362	7.383754513
**592**	1.259995319	7.395604162
**593**	1.262138298	7.404969208
**594**	1.264268085	7.416210094
**595**	1.266395745	7.425893679
**596**	1.268524681	7.437920294
**597**	1.270655319	7.449026686
**598**	1.272792766	7.460925887
**599**	1.274922553	7.470651943
**600**	1.277049362	7.48559496
**601**	1.27918383	7.497508317
**602**	1.281314894	7.510341898
**603**	1.283451064	7.520252
**604**	1.285575319	7.530055921
**605**	1.287705106	7.540440292
**606**	1.289845957	7.551851065
**607**	1.291974894	7.563198131
