# Supplementary material for: Design and optimization of soft finger actuators for rehabilitation applications: A combined finite element and neural network approach
Source: PLoS One. 2025 Oct 31;20(10):e0334011. doi: 10.1371/journal.pone.0334011 (PMC12578219; doi:10.1371/journal.pone.0334011)
Supplement: Results models at 2 mm — This table presents the relationship between the load and the deformation on the X-axis, and the Y-axis at different cases for the Yeoh second order, Yeoh third order, Mooney Rivlin (second, Third, and Fifth) orders. (PDF) [file pone.0334011.s002.pdf]

#	P1 - Solid Assignment	P2 - X Directional Deformation Minimum [mm]	P4 - X Directional Deformation Maximum [mm]	P5 - Y Directional Deformation Minimum [mm]	P6 - Y Directional Deformation Maximum [mm]
**Name**	**P1**	**P2**	**P4**	**P5**	**P6**
DP 0	TPU Yeoh 2nd	-0.713726521	0.719455361	-76.64097595	2.390338421
DP 1	TPU Yeoh 3rd order	-0.615535498	0.618697345	-82.12664795	1.9710114
DP 2	Mooney-Rivlin 2 Parameter	-0.117115989	0.115210004	-61.6331749	0.191363007
DP 3	Mooney-Rivlin 3 Parameter	-0.076506414	0.076573372	-40.78669739	0.118119456
DP 4	Mooney-Rivlin 5 Parameter	-0.045725	0.046276	-22.661	0.066582

#	P1 - Solid Assignment	P7 - Z Directional Deformation Minimum [mm]	P8 - Z Directional Deformation Maximum [mm]	P10 - Total Deformation Maximum [mm]	P11 - Equivalent Elastic Strain Minimum [mm mm^-1]
**Name**	**P1**	**P7**	**P8**	**P10**	**P11**
DP 0	TPU Yeoh 2nd	-175.4345703	7.82793951	178.3845364	0.000191153
DP 1	TPU Yeoh 3rd order	-175.0478668	7.265148163	184.0762188	0.000163655
DP 2	Mooney-Rivlin 2 Parameter	-19.30256462	2.870530605	63.73751131	9.83E-06
DP 3	Mooney-Rivlin 3 Parameter	-8.338092804	1.980272532	41.31652267	6.15E-06
DP 4	Mooney-Rivlin 5 Parameter	-2.6855	1.154	22.737	5.50E-06

#	P1 - Solid Assignment	P12 - Equivalent Elastic Strain Maximum [mm mm^-1]	P13 - Equivalent Stress Minimum [MPa]	P14 - Equivalent Stress Maximum [MPa]
**Name**	**P1**	**P12**	**P13**	**P14**
DP 0	TPU Yeoh 2nd	0.636054504	0.000159299	3.872948942
DP 1	TPU Yeoh 3rd order	0.496809974	8.63E-05	3.948871907
DP 2	Mooney-Rivlin 2 Parameter	0.125118303	5.96E-05	2.56124063
DP 3	Mooney-Rivlin 3 Parameter	0.081647752	5.35E-05	2.667487769
DP 4	Mooney-Rivlin 5 Parameter	0.044351	2.31E-04	2.7552
