# Supplementary material for: Design and optimization of soft finger actuators for rehabilitation applications: A combined finite element and neural network approach
Source: PLoS One. 2025 Oct 31;20(10):e0334011. doi: 10.1371/journal.pone.0334011 (PMC12578219; doi:10.1371/journal.pone.0334011)
Supplement: Attachment — Submitted filename: Comments.pdf [file pone.0334011.s003.pdf]

This study presents a detailed analysis of soft finger actuators for rehabilitation gloves using finite element modeling and neural networks. It explores how design parameters like bellows number and actuator height affect actuator bending, optimizing performance for therapeutic use. Experimental validation confirms the model's high predictive accuracy. Increasing the bellows height enhances bending angles, while thicker materials decrease flexibility, emphasizing the importance of precise design in developing effective rehabilitation technologies. However, despite its progress, significant concerns remain unaddressed. Some of the raised concerns are mentioedn below:

1. **Lack of Comparisons with Existing Technologies:** While the manuscript thoroughly investigates the proposed design, it lacks a comparative analysis with existing technologies. Including such comparisons could significantly enhance the relevance and impact of the findings by positioning this research within the larger field of soft robotics.
2. **Potential Overfitting in Neural Network Modeling:** The high accuracy of the neural network model is impressive; however, the risk of overfitting should be addressed. It would be beneficial for the authors to discuss the steps taken to prevent overfitting and to ensure the model's generalizability to other similar applications.
3. **Limited Discussion on Material Choices:** The choice of materials, particularly silicone rubber, is briefly discussed in terms of its mechanical properties. However, a more detailed rationale for choosing this material over others, considering the specific requirements of rehabilitation applications, would strengthen the manuscript.
4. **Expand on Material Selection Criteria:** Provide a more detailed analysis of why silicone rubber was chosen over other potential materials, including an assessment of its performance, durability, and safety in contact with skin.
5. **Incorporate Comparative Studies:** Add a section comparing the newly designed actuators with existing products or research in the field of soft robotics to highlight the advancements your design provides.
6. **Address Model Generalization:** Enhance the discussion on the neural network's generalization capabilities by including tests on external datasets or through cross-validation techniques.
7. References: The following refeercnes are highly recommended toa dded to the paper to improve its quality and state of the arts. (10.1007/s41315-025-00421-x), (10.22024/UniKent/01.02.105160), and (10.1109/ACCESS.2023.3325211).
