# Supplementary material for: Design and optimization of soft finger actuators for rehabilitation applications: A combined finite element and neural network approach
Source: PLoS One. 2025 Oct 31;20(10):e0334011. doi: 10.1371/journal.pone.0334011 (PMC12578219; doi:10.1371/journal.pone.0334011)
Supplement: Attachment — Submitted filename: Reviewers Reply.pdf [file pone.0334011.s004.pdf]

Dear POLS ONE Editor

Dear Reviewer #1,

We are pleased to submit the revised version of our manuscript entitled " **Design and Optimization of Soft Finger Actuators for Rehabilitation Applications: A Combined Finite Element and Neural Network Approach**" for your review. We have considered all of the comments provided and made significant revisions to improve the quality of our work.

In response to Reviewer #1's comments, we have thoroughly revised the manuscript to address each point in a concrete, concise, and direct manner. Specifically:

1. **Point 1: Lack of Comparisons with Existing Technologies: While the manuscript thoroughly investigates the proposed design, it lacks a comparative analysis with existing technologies. Including such comparisons could significantly enhance the relevance and impact of the findings by positioning this research within the larger field of soft robotics.**

Point 1 Reply: We sincerely thank you for this constructive comment. We agree that positioning our contribution within the broader landscape of soft robotic actuators is essential to underscore its relevance and impact. In response, we have (i) expanded our comparative analysis with representative state-of-the-art technologies and (ii) incorporated new text and quantitative benchmarks into the manuscript. Below, we summarize the substantive additions and specify precisely where new content has been integrated.

1. Summary of our additions

- Expanded Related Work and Contextualization:
    - We enhanced the background discussion to explicitly compare our segmented, low-pressure, monolithic soft finger actuator against benchmark families: PneuNets, fiber-reinforced bending actuators, and McKibben muscles, highlighting differences in operating pressure, bending angle, ergonomics, manufacturability, and control complexity.
- Standardized, quantitative comparisons:
    - We added comparative performance curves and normalized metrics discussing bending angle versus pressure, response time, and fabrication complexity, and we contextualized our results at the same nominal pressure range where possible.
- Discussion of rehabilitation-centric criteria:

- We emphasized criteria that are particularly relevant to rehabilitation settings (safety at sub-100 kPa, ergonomic segmentation aligned with MCP–PIP–DIP, single-input control for clinical simplicity). We contrasted these with trade-offs commonly found in higher-pressure or more complex actuator architectures.

2. Technical rationale for our approach in the context of existing technologies

- Pressure and safety envelope:
  - Many widely used soft actuators (e.g., fiber-reinforced and McKibben types) achieve high force or rapid contraction but typically require higher pressures (≥150–500 kPa), which increases pneumatic system complexity and may raise safety and comfort concerns in patient-facing devices. In contrast, our design demonstrates large bending angles at sub-100 kPa, reducing hardware burden and improving clinical viability.
- Ergonomics and segmentation:
  - Unlike general-purpose continuum soft actuators, our architecture is segmented to anatomically mirror the MCP–PIP–DIP joints, improving glove integration, joint-specific assistance, and fit across hand morphologies.
- Manufacturability and customization:
  - Our monolithic, 3D-printed design supports rapid, patient-specific customization without multi-layer assembly or fiber winding, reducing variability and easing clinical translation.

3. New text inserted into the manuscript

To ensure the manuscript itself addresses your request, we have inserted the following lines. For clarity, we provide the exact passages and their intended locations.

A) End of "Modeling of Soft Finger Actuators" section
New paragraph:

"Comparative Positioning within State-of-the-Art Soft Actuators. To contextualize the proposed actuator within the broader field, we contrast its performance and design attributes against three representative families: PneuNets, fiber-reinforced bending actuators, and McKibben muscles. As summarized in Table 1, our actuator achieves a maximum bending angle of 115° at 90 kPa, while operating within a sub-100 kPa envelope that is attractive for rehabilitation safety and system simplicity. PneuNets often require up to 120–200 kPa to reach comparable deformations and involve multi-layer molding, whereas fiber-reinforced actuators can deliver high forces but typically at ≥150 kPa with increased control complexity due to pressure sequencing and anisotropic reinforcements. McKibben muscles provide large linear forces but are not inherently joint-conformal and

generally demand higher operating pressures (≥300–500 kPa). In contrast, our segmented, anatomically inspired architecture combines large bending at lower pressure, monolithic fabrication for rapid patient-specific customization, and low control complexity (single pneumatic input), thereby addressing clinically relevant constraints for hand rehabilitation."

B) **After Table 1 (same section), we add a concise analysis paragraph that links quantitative entries to design choices:**
New paragraph:
"Interpreting the Comparison. The observed performance advantages at lower pressure are attributable to: (i) the segmented, joint-mimetic geometry that concentrates volumetric expansion where bending moments are maximized; (ii) the tuned balance between wall thickness (TSB) and foot thickness (TOF), which reduces unnecessary stiffness without compromising structural integrity; and (iii) monolithic 3D printing that preserves chamber fidelity and minimizes leakage at interfaces. These design choices jointly enable higher bending per unit pressure, which is advantageous in clinical environments that favor compact, low-pressure pneumatic hardware."

C) **In "FEM Results Discussions" (end of the section), we add a paragraph to explicitly compare our parametric trends with prior reports:**
New paragraph:
"Consistency with and Differentiation from Prior Actuators. Our parametric trends—namely that increasing chamber height (H) and the number of chambers (N) increase bending, whereas increasing surrounding thickness (TSB) and foot thickness (TOF) decrease bending—are consistent with foundational findings in soft pneumatic architectures (e.g., PneuNet-style chambers and fiber-reinforced geometries). However, the segmented, finger-joint-centric configuration herein yields these gains at lower operating pressures, enabling clinically desirable deformation without resorting to high-pressure infrastructure or complex reinforcement schemes. This represents a distinct positioning relative to general-purpose soft actuators primarily optimized for maximal force or workspace at higher pressures."

D) **In "Experimental Test and Validation" (end of section), we add a statement that frames our results under comparable operating conditions:**
New paragraph:
"Operating Envelope Consideration. While diverse soft actuators can achieve large deformations, we emphasize that our experimental validation targets a sub-100 kPa envelope consistent with rehabilitation safety guidelines and portable pneumatic systems. Under this constraint, the measured bending angles align favorably with or exceed those reported for comparable chambered designs operated at higher pressures, reinforcing the suitability of the proposed actuator for clinical hand-assist use cases."

Point 2: Potential Overfitting in Neural Network Modeling: The high accuracy of the neural network model is impressive; however, the risk of overfitting should be addressed. It would be beneficial for the authors to discuss the steps taken to prevent overfitting and to ensure the model's generalizability to other similar applications.

Point 2 Reply: We appreciate this important observation regarding potential overfitting in our neural network (NN) modeling. Ensuring generalizability beyond the training set is central to our goal of providing a reliable surrogate for design exploration. In response, we (i) clarify the safeguards against overfitting already used, (ii) report additional analyses, and (iii) add explicit methodological details and limitations in the manuscript. Below, we summarize our technical position and provide text insertions.

1. Technical response: measures taken to mitigate overfitting and assess generalization

- Data provenance and coverage of design space
    - The NN was trained primarily on a systematic, FEM-generated design-of-experiments (DoE) grid spanning the four geometric variables: Height (H), Number of Bellows (N), Thickness Surrounding Bellows (TSB), and Thickness of Foot (TOF), at the operating pressure used in our validation studies. This grid-based sampling reduces selection bias and provides uniform coverage within the targeted design bounds, thus lowering the risk of overfitting to localized clusters.
- Train/validation/test protocol and early stopping
    - We split the dataset into training (70%), validation (15%), and test (15%) sets using a stratified scheme across the four inputs to preserve the distribution of H, N, TSB, and TOF. Training employed early stopping on validation loss with patience to prevent over-training.
- Regularization and normalization
    - We used L2 weight regularization on dense layers and input feature standardization (z-scoring) to stabilize optimization and reduce variance. Batch size and learning rate were tuned to avoid memorization effects.
- Externalization via holdout “fabrication set” checks
    - Although the network was trained on FEM data, we evaluated generalization by comparing NN predictions against experimental bending angles for fabricated samples at multiple configurations not used for model tuning. The reported agreement in Figures 20–24 reflects this sim-to-real validation step, which serves as a preliminary external test set.

- Error analysis and calibration
    - We examined test residuals across the input domain and did not observe systematic bias at the design-space boundaries. The scatter around the identity line (Fig. 22) further supports the model's calibration. We now report per-region errors (see added text below).

2. New text inserted into the manuscript

   In "Neural Network Modeling" (Methods):
   New paragraph:

"Overfitting Mitigation and Validation Protocol. To reduce the risk of overfitting and enhance generalizability, we adopted the following safeguards: (i) a grid-based design-of-experiments (DoE) spanning the four geometric inputs (H, N, TSB, TOF) to ensure broad and uniform coverage of the design space; (ii) stratified splitting into training (70%), validation (15%), and test (15%) subsets that preserved the marginal distributions of all inputs; (iii) input feature standardization (z-score) and L2 weight regularization on dense layers; and (iv) early stopping based on validation loss with a patience criterion to prevent over-training. We constrained model capacity to the smallest architecture that achieved validation convergence, avoiding excessively deep or wide networks that may memorize training samples. To preliminarily assess sim-to-real transfer, we also compared NN predictions to experimental measurements from a set of fabricated actuators not used during model tuning (see Figs. 20–24)."

Point 3: Limited Discussion on Material Choices: The choice of materials, particularly silicone rubber, is briefly discussed in terms of its mechanical properties. However, a more detailed rationale for choosing this material over others, considering the specific requirements of rehabilitation applications, would strengthen the manuscript.

Point 4: Expand on Material Selection Criteria: Provide a more detailed analysis of why silicone rubber was chosen over other potential materials, including an assessment of its performance, durability, and safety in contact with skin.

Point 3 and 4 Reply: We appreciate this insightful comment. We agree that a more explicit, application-driven rationale for the material selection will strengthen the manuscript, especially given the safety and comfort requirements of rehabilitation. In response, we (i) expand the justification for silicone rubber as the primary elastomer, (ii) contextualize it against alternative materials (e.g., TPU, Ecoflex/Pt-cured silicones of varying shore hardness,

urethanes, and fiber-reinforced composites), and (iii) insert new text into the manuscript to clearly connect material properties to clinical and engineering constraints.

1. Technical rationale for selecting silicone rubber in rehabilitation-oriented SPAs

- Biocompatibility and skin contact safety:
    - Medical/skin-contact grade platinum-cured silicones exhibit low cytotoxicity, are hypoallergenic, and can be formulated to meet ISO 10993 biocompatibility pathways, making them suitable for prolonged dermal contact typical of hand rehabilitation sessions.
- Tunable compliance and low durometer:
    - Silicones are available across a wide range of Shore A (e.g., A00–A40+), enabling selection of a modulus window that achieves large, low-pressure bending while maintaining comfort and conformity to hand anatomy. This supports our sub-100 kPa operating envelope and reduces the need for bulky pneumatic hardware.
- Hyperelastic stability and model fidelity:
    - Silicones show well-characterized, repeatable hyperelastic behavior over large strains, allowing accurate fitting with Yeoh/Ogden models. This underpins the close FEM–experiment agreement reported and supports predictive design and ANN training.
- Processing, sealing, and monolithic fabrication:
    - Castable/printable silicones enable monolithic chamber fabrication with excellent interlayer adhesion and low permeability at interfaces, minimizing leakage. They cure at room or moderate temperatures, facilitating rapid iteration and patient-specific customization.
- Hysteresis and fatigue:
    - Compared to some urethanes and TPUs, silicone rubbers often exhibit lower mechanical hysteresis and good elastic recovery under cyclic loading relevant to repetitive rehabilitation tasks. While fatigue life depends on formulation and geometry, silicone's damage progression is gradual and detectable, aiding preventive maintenance.
- Thermal and chemical stability:
    - Silicones maintain properties across typical clinical temperature/humidity ranges, are resistant to sweat/saline, and tolerate gentle disinfection protocols without embrittlement—important for hygiene in multi-user settings.

2. New text to be inserted into the manuscript

A) In "Hyper-Elastic Material Characterization" (end of section):
New paragraph

"Material Selection Rationale for Rehabilitation Context. We selected a platinum-cured silicone rubber as the primary elastomer due to its favorable combination of skin-contact safety, tunable compliance, and stable hyperelastic response under large deformations. Medical-grade silicone formulations are widely used in dermal-contact applications and can be qualified along ISO 10993 pathways, aligning with hygiene and safety requirements in clinical environments. From a mechanics standpoint, silicone's low-to-moderate Shore A hardness and low hysteresis enable large bending at sub-100 kPa with rapid elastic recovery—critical for comfortable, repetitive motion assistance. Processing advantages (room-temperature curing, strong interlayer adhesion, and low interface permeability) facilitate monolithic chamber fabrication with reduced leakage risk, supporting the patient-specific customization central to rehabilitation. Alternative elastomers such as TPUs and urethanes offer higher tear resistance but typically require increased thickness or higher pressures to achieve similar bending, which counters our low-pressure design objective and may reduce comfort. Fiber-reinforced laminates can increase force output but introduce stiffness anisotropy and fabrication complexity, lessening ergonomic compliance. Thus, the chosen silicone balances safety, compliance, manufacturability, and modeling fidelity, which collectively underpins the observed agreement between FEM predictions and experiments."

B) In "Modeling of Soft Finger Actuators":
New paragraph:

"The segmented, joint-mimetic geometry is intentionally paired with a low-durometer silicone to concentrate volumetric expansion at the bending loci and achieve large angular excursions at sub-100 kPa. This material–architecture synergy reduces pneumatic hardware burden and enhances user comfort by maintaining a soft, conformal interface with minimal localized pressure on the skin."

Point 5: Incorporate Comparative Studies: Add a section comparing the newly designed actuators with existing products or research in the field of soft robotics to highlight the advancements your design provides.

Point 5 Reply: We appreciate this valuable recommendation. We agree that an explicit, standardized comparison with representative soft actuator families will better contextualize our contribution and highlight where our design advances the state of the art, particularly under the rehabilitation-driven constraint of sub-100 kPa operation. In response, we have: (i) added a dedicated comparative section with normalized metrics; (ii) inserted a quantitative table contrasting our actuator against PneuNets, fiber-reinforced bending actuators, and McKibben muscles; and (iii) expanded the discussion to interpret mechanism-level reasons for the observed performance differences. Below we summarize the additions and provide verbatim text with insertion points.

1. Technical rationale and scope of comparison

- Why these comparators:
    - PneuNets, fiber-reinforced bending actuators, and McKibben muscles are widely used baselines in soft robotics and cover the dominant paradigms for bending and linear artificial muscle actuation.
- Normalization and relevance:
    - We focus on rehabilitation-relevant criteria: bending angle per unit pressure (efficiency under sub-100 kPa), response time, ergonomics/joint conformity, control complexity, and fabrication method. Where possible, we compare at overlapping operating pressures and report geometry/context to avoid unfair advantages.
- Mechanistic explanation:
    - We interpret differences through geometry/material coupling, stiffness distribution (TSB/TOF), and architectural segmentation that mirrors MCP–PIP–DIP joints, which together enable large bending at lower pressures and simpler control.

2. New manuscript content (verbatim text and locations)

A) New subsection in "Modeling of Soft Finger Actuators" (after current Figs. 2–3 and before "Several geometric dimensions …")
New subsection title and text:

"Although soft pneumatic actuators have been widely explored in robotics, existing designs often prioritize general-purpose functionality over the specialized demands of hand rehabilitation. For context, we compare the proposed segmented, monolithic actuator against three representative families: PneuNets, fiber-reinforced bending actuators, and McKibben muscles. Table 1 summarizes key metrics under comparable operating conditions. Our actuator achieves a maximum bending angle of 115° at 90 kPa, emphasizing efficient deformation within a sub-100 kPa envelope that aligns with clinical safety and portable pneumatic hardware. PneuNet actuators commonly require 120–200 kPa for similar angular excursions and depend on multi-layer molding. Fiber-reinforced actuators can deliver higher directional force but typically operate at ≥150 kPa and involve increased control and fabrication complexity due to anisotropic reinforcement. McKibben muscles provide high linear force but are not inherently joint-conformal and commonly require 300–500 kPa. The proposed design combines: (i) joint-mimetic segmentation that concentrates volumetric expansion at bending loci, (ii) tuned wall (TSB) and foot (TOF) thicknesses that avoid unnecessary stiffness, and (iii) monolithic 3D-printed fabrication that preserves chamber fidelity and minimizes leakage. These factors jointly enable higher bending per unit pressure with low control complexity (single pneumatic input), enhancing suitability for hand rehabilitation."

B) Immediately following Table 1, we add an interpretive paragraph to emphasize standardized and normalized comparisons:
New paragraph:

"Standardized Comparison and Normalization. To reduce bias across platforms, we emphasize comparisons within overlapping pressure ranges and normalize bending efficiency by reporting angle at sub-100 kPa where available. While exact actuator geometries differ, the proposed design's segmented architecture and low-durometer silicone consistently increase bending per unit pressure relative to chambered and fiber-reinforced baselines operated above 120–150 kPa. Where direct force normalization is limited by architectural differences (e.g., McKibben's linear contraction), we contextualize rehabilitative relevance

through ergonomic conformity, control simplicity, and safety envelope, which are critical determinants for clinical deployment."

Point 6: Address Model Generalization: Enhance the discussion on the neural network's generalization capabilities by including tests on external datasets or through cross-validation techniques.

Point 6 Reply: Thank you for this important point. We have already discussed and presented the neural network's generalization in detail in the manuscript:

- Neural Network Modeling section: We describe the train/validation/test protocol, overfitting mitigation (L2 regularization, z-score normalization, early stopping), and the standardized data split ensuring generalization beyond the training subset.
- Overfitting Mitigation and Validation Protocol subsection: We outline the design-of-experiments coverage across H, N, TSB, and TOF, and the validation procedure used to assess model robustness.
- Experimental Test and Validation section (Figs. 20–24): We provide sim-to-real evaluation by comparing ANN predictions with measurements from fabricated actuators not used in model tuning, demonstrating external generalization under the sub-100 kPa operating envelope.
- Results around Fig. 22–24: We report high explained variance (99.26%) and low residual variance (0.74%), along with close alignment between predicted and measured bending angles, evidencing robust generalization within the studied design bounds.

Point 7: References: The following references are highly recommended to be added to the paper to improve its quality and state of the art. (10.1007/s41315-025-00421-x), (10.22024/UniKent/01.02.105160), and (10.1109/ACCESS.2023.3325211).

Point 7 Reply: Thank you for these constructive suggestions. We agree that incorporating the recommended references will strengthen the manuscript's coverage of the state of the art. We have added the three works to the References and cited them at contextually relevant points in the text.

Throughout the revision process, I carefully considered all the comments and suggestions provided by Reviewer #1 and have made every effort to address each thoroughly and meaningfully. We believe that the changes we have made will significantly improve the manuscript and address any concerns that were raised.

I'm confident that the revised version of our manuscript represents a significant improvement over the original submission, and we are grateful for the opportunity to resubmit it for your consideration.

Thank you for your time and consideration.

Sincerely,

Dear POLS ONE Editor

Dear Reviewer #2,

We are pleased to submit the revised version of our manuscript entitled " **Design and Optimization of Soft Finger Actuators for Rehabilitation Applications: A Combined Finite Element and Neural Network Approach**" for your review. We have considered all of the comments provided and made significant revisions to improve the quality of our work.

In response to Reviewer #2's comments, we have thoroughly revised the manuscript to address each point in a concrete, concise, and direct manner. Specifically:

1. **Point 1: The introduction discusses the importance of rehabilitation technologies and soft actuators, but it lacks a clear research gap. The authors should explicitly state what existing limitations in soft actuator design their study addresses.**

Point 1 Reply: Thank you for this constructive comment. We agree that the Introduction benefits from an explicit statement of the research gap and our contributions relative to limitations in current soft actuator designs for rehabilitation. In response, we have (i) clarified the unmet needs that motivate our work, (ii) articulated the specific limitations of existing soft actuator families under rehabilitation-relevant constraints, and (iii) stated our contributions in a concise gap-and-contribution paragraph. Below, we provide a technical rationale and the exact manuscript insertions.

1. Technical rationale: what gap we address

- Low-pressure, rehabilitation-centric operation: Many established soft actuators (e.g., PneuNets, fiber-reinforced, McKibben) achieve large deformation or high force but often at pressures ≥120–150 kPa (and up to 500 kPa), which increases hardware burden and safety risk for portable, home-based hand therapy. The gap is a design that achieves large, controllable bending at sub-100 kPa while remaining ergonomic and easy to control.
- Ergonomic joint conformity and simplified control: General-purpose actuators may not be anatomically segmented, leading to poorer joint conformity, higher localized pressures on the skin, or the need for multi-channel control and pressure sequencing. The gap is an actuator architecture that maps to MCP–PIP–DIP joints, provides a conformal interface, and operates with a single pneumatic input.
- Predictive, design-space-efficient modeling: Literature often reports either FEM-only exploration or empirical tuning. There is limited integration of validated FEM with a data-driven surrogate capable of accurately predicting bending from key geometric parameters to accelerate iterative design under clinical constraints. The gap is a combined FEM–ANN

pipeline validated against experiments and suitable for rapid, low-pressure design optimization.

- Rehabilitation-specific benchmarking: Comparative studies frequently emphasize force/workspace without normalizing for low-pressure operation or ergonomic conformity. The gap is a standardized comparison at overlapping pressures, reporting bending-per-pressure, response time, ergonomics, and control complexity in a rehabilitation context.

2. **Additions to the manuscript**

   A) **Introduction section — we added the following paragraph at the end of the final paragraph of the Introduction:**

"Research Gap and Study Objectives. Despite recent advances in soft actuation for manipulation and mobility, three limitations persist for hand rehabilitation: (i) many actuators achieve their reported bending or force output at ≥120–150 kPa (and up to 500 kPa), which burdens portable hardware and raises safety concerns for prolonged, home-based use; (ii) general-purpose designs often lack anatomical segmentation and joint conformity, increasing localized skin pressure and control complexity (e.g., multi-channel pressure sequencing); and (iii) predictive design tools that couple validated FEM with efficient, data-driven surrogates remain underutilized for rapid, low-pressure optimization. Addressing these gaps, this work develops a segmented, anatomically inspired soft finger actuator that delivers large, controllable bending at sub-100 kPa with a single pneumatic input, and establishes a combined FEM–ANN pipeline—validated experimentally—to accurately predict bending from four key geometric parameters (H, N, TSB, TOF). We further provide a rehabilitation-centric comparison against representative actuator families, normalized by operating pressure, to contextualize the design's advantages in safety, ergonomics, and control simplicity."

Point 2: The motivation behind using ANNs should be elaborated. Why was ANN chosen over other modeling techniques? How does it compare to conventional regression models or other machine learning approaches?

Point 2 Reply: Thank you for this valuable suggestion. We agree that the manuscript should more clearly articulate the rationale for selecting artificial neural networks (ANNs), and position them against conventional regression and alternative machine learning approaches under our problem constraints.

1. Technical rationale for choosing ANNs in this study

- Nonlinear, interaction-rich design space: The mapping from geometric parameters (H, N, TSB, TOF) to bending angle is strongly nonlinear and exhibits interactions (e.g., the marginal effect of H depends on N and TSB/TOF). Our FEM results and multi-parameter plots (Figs. 8–15) reveal monotonic trends with saturation and interaction effects that are not well captured by linear or low-order polynomial regressions without extensive feature engineering and risk of extrapolation artifacts.

- Smooth interpolation within bounded domain: Our target use-case is fast, accurate interpolation within a bounded, well-sampled design space for low-pressure (≤90 kPa) operation, not extrapolation far beyond it. Feedforward ANNs with ReLU activations provide flexible function approximation with smoothness that we regulate through capacity control (restricted width/depth), L2 regularization, early stopping, and standardized inputs. This balances accuracy and generalization within the design bounds.

- Data efficiency with FEM-augmented training: Compared to high-variance nonparametric methods (e.g., unrestricted kernel regressions), compact ANNs can learn efficient internal representations from FEM-generated data with fewer hyperparameters to tune, and they deploy quickly for design iteration. They serve as a lightweight surrogate for rapid design screening before resorting to new FEM runs.

- Robustness vs. simpler baselines: We evaluated simpler baselines (linear and polynomial regression up to degree 3, not included previously for brevity) and found noticeable underfit at mid-to-high H and N, and systematic bias when TSB/TOF increased (consistent with nonlinear stiffness effects). Tree-based models (e.g., random forests, gradient boosting) can achieve low training error but often produce piecewise-constant response surfaces with limited smoothness, which is undesirable for gradient-like design navigation and can overfit in sparsely sampled corners. Gaussian Processes (GPs) offer uncertainty estimates but scaled poorly with our dataset size and required careful kernel design to accommodate mixed-scale inputs; preliminary trials showed comparable accuracy but higher computational overhead and sensitivity to kernel choices.

- Practicality for rehabilitation-centric pipelines: We required a fast, easily integrable, and reproducible surrogate that runs on modest hardware, supports real-time what-if analysis, and yields stable predictions under standardized preprocessing. The implemented ANN met these constraints while achieving very low residual variance (0.74%) and high explained variance (99.26%) on held-out data, and it generalized to fabricated actuators under sub-100 kPa conditions (Figs. 20–24).

2. Where this is already covered and how we will strengthen it

- Existing relevant section: “Neural Network Modeling” and “Overfitting Mitigation and Validation Protocol” already outline our data sources, inputs/outputs, regularization (L2), early stopping, standardization, and the stratified splits with sim-to-real checks. However, they do not explicitly compare ANNs to alternative models or justify the selection.

Point 3: The choice of thermoplastic polyurethane as the material for the actuator should be justified. Are there any comparisons with other materials in terms of elasticity, fatigue resistance, or biocompatibility?

Point 3 Reply: Thank you for highlighting the need to justify our actuator material choice and to contextualize it against alternatives. We appreciate this opportunity to clarify. In the current manuscript, the "Hyper-Elastic Material Characterization" section describes our experimental material testing and explicitly states that the actuators used in this study were fabricated from a platinum-cured silicone rubber, selected for its compliance, skin-contact safety, and stable hyperelastic response under large deformations. TPU is mentioned in the keywords due to its prevalence in soft actuator literature and our broader design considerations; however, the presented FEM, experiments, and ANN results pertain to silicone-based actuators. We recognize that this may have caused confusion and have revised the text to make our choice and the rationale unambiguous, while adding a concise comparison to TPU and related elastomers.

Technical rationale based on elasticity, fatigue/durability, and biocompatibility

- Elasticity and low-pressure bending efficiency
    - Our rehabilitation-centric constraint is sub-100 kPa operation with large, controllable bending. Platinum-cured silicones with low-to-moderate Shore A hardness typically exhibit lower hysteresis and higher recoverable strain at a given pressure than comparable-thickness TPU films, enabling larger bending per unit pressure. This aligns with our FEM trends and experiments showing 115° at 90 kPa.
    - The Yeoh 3rd-order fit we report (Hyper-Elastic Material Characterization) captures a stable, smooth stress–strain curve suitable for large deformation modeling and closely matches measured bending. This modeling fidelity is critical to our FEM–ANN pipeline.
- Fatigue resistance and durability
    - For cyclic rehabilitation use, both tear resistance and hysteresis matter. TPU is known for high tear strength and abrasion resistance, often favored where thin membranes or high-cycle pressurization are required. However, TPUs of comparable stiffness can demand higher thickness or operating pressure to achieve the same bending, which conflicts with our sub-100 kPa envelope.
    - Platinum-cured silicones used here offer low mechanical hysteresis and elastic recovery conducive to repetitive actuation at low pressure. While absolute tear resistance of TPU may exceed that of silicone for thin films, our monolithic, chambered silicone design mitigates stress risers and leakage-prone interfaces, and our FEM-guided geometry avoids sharp curvature concentrations, supporting durability for the intended loading.

- Biocompatibility and skin contact
    - Platinum-cured silicones are widely used in dermal-contact applications and can be qualified under ISO 10993 pathways. This aligns with clinical and home-use rehabilitation needs. Many TPUs are also biocompatible, but require careful grade selection, plasticizer/additive control, and bonding/assembly considerations to ensure long-term skin safety and stability. Our selection emphasizes a clinically familiar, low-irritation interface with proven use in medical wearables.

Where this fits in the manuscript and new insertions

Existing relevant section:

- "Hyper-Elastic Material Characterization" already explains the choice of a platinum-cured silicone for safety, compliance, manufacturability, and modeling fidelity. To directly address your comment, we add a brief, explicit comparison to TPU and clarify why silicone was preferred for this study's constraints.

Point 4: The rationale for the selection of geometric parameters should be further explained. Why were these specific values chosen for the number of bellows, height, and thicknesses? Did preliminary studies influence these choices?

Point 4 Reply: Thank you for this insightful comment. We agree that making our rationale for the selected geometric parameter ranges more explicit will strengthen the manuscript. Below, we (i) provide a technical justification for the ranges chosen for the number of bellows (N), bellow height (H), surrounding wall thickness (TSB), and foot thickness (TOF); (ii) clarify how preliminary studies, anatomical/ergonomic constraints, and fabrication limits informed these choices; and (iii) propose verbatim additions to the manuscript to document this rationale in-place.

1. Technical rationale and constraints that shaped the chosen ranges

- Rehabilitation-driven kinematics at sub-100 kPa:
    - Our target was reliable, joint-mimetic bending sufficient to support grasp-like motions with ≤90 kPa supply, to maintain portability and safety. This objective dictated a design space emphasizing bending-per-pressure efficiency rather than peak force. The ranges of H, N, TSB, and TOF were selected to span practical trade-offs between compliance and structural integrity within this pressure envelope.
- Anatomical and ergonomic constraints:
    - The total actuator length (~130 mm) maps to the combined length of the index finger phalanges plus joint spacing for adult users, with segmented actuation to mimic MCP–PIP–DIP. Given the chamber pitch and required neutral spacing for compliant folding, the feasible N range was bounded so that the chamber count

would not exceed the anthropometric envelope nor yield subcritical chamber widths that are difficult to print and seal.

- Fabrication and sealing constraints (monolithic 3D-printed silicone):
  - Minimum printable and reliable wall thicknesses and features were empirically validated through pilot prints. TSB values below ~1.0 mm increased risk of local buckling and leakage; above ~1.75–2.0 mm, bending became overly stiff at ≤90 kPa. Similarly, TOF below ~1.25 mm raised risks of footer distortion and delamination at the interface with the passive layer, whereas TOF above 2.0 mm significantly suppressed bending without commensurate gains in robustness.
  - For H, a span of 10–25 mm captured the monotonic gains in bending angle with diminishing returns near 25 mm, while staying within the form factor of a wearable glove and preserving chamber stability under inflation.
- Preliminary simulations and benchtop pilots:
  - Prior to the full factorial FEM study, we conducted screening simulations and built pilot actuators to verify printability, sealing, and bending at ≤90 kPa. These pilots showed:
    - H < 10 mm produced limited bending (<80° at 90 kPa) with higher sensitivity to TSB/TOF tolerances.
    - H > 25 mm began to suffer from local wrinkling and out-of-plane deformation unless TSB increased, which defeated the sub-100 kPa objective.
    - N in the [12, 20] range enabled a good distribution of curvature over the anthropometric length; N < 12 yielded insufficient angular excursion, while N > 20 compressed chamber spacing below printable limits with our process and increased flow resistance.
    - TSB in [1.0, 1.75] mm and TOF in [1.25, 2.0] mm formed a practical window balancing stiffness, durability, and bending-per-pressure.
  - These findings directly shaped the final design-of-experiments grid reported in Table 2 and the FEM analyses in Figs. 8–15, which then informed the ANN training set.

2. Where this already appears and how we will strengthen it

- Existing relevant sections:
  - Modeling of Soft Finger Actuators: establishes ergonomic/anthropometric motivation and joint-mimetic segmentation.
  - Finite Element Analysis and FEM Mesh Generation: detail modeling and boundary conditions but do not yet state the parameter-selection rationale explicitly.

- FEM Results Discussions: analyze parametric effects but do not explain why these specific ranges were chosen.

- We propose adding a concise "Parameter Selection Rationale" subsection in System Modeling > Modeling of Soft Finger Actuators, and a brief note in Finite Element Analysis to link the ranges to the low-pressure operating envelope and fabrication constraints.

3. Verbatim additions to the manuscript

A) We insert at the end of "Modeling of Soft Finger Actuators:

"Parameter Selection Rationale for Rehabilitation Context. The geometric ranges investigated in this study were bounded by three practical considerations: (i) rehabilitation-driven kinematics at sub-100 kPa, (ii) anatomical conformity to finger segment lengths, and (iii) fabrication and sealing reliability in monolithic, 3D-printed silicone. Preliminary screening (FEM and benchtop pilots) indicated that bellow heights H below 10 mm yielded limited bending and high sensitivity to wall tolerances, whereas H above 25 mm introduced local wrinkling or out-of-plane deformation unless wall thicknesses increased, which conflicted with our low-pressure objective. The number of bellows N was confined to 12–20 to distribute curvature over a ~130 mm actuator length without reducing chamber pitch below printable limits or increasing pneumatic resistance; $N < 12$ underperformed in angular excursion, and $N > 20$ compromised printability and sealing. The surrounding wall thickness TSB was limited to 1.0–1.75 mm to avoid leakage/buckling at the low end and undue stiffness at the high end; the foot thickness TOF was set to 1.25–2.0 mm to balance bending efficiency with structural support and interface stability. These empirically validated bounds defined the design-of-experiments grid used for FEM sweeps and ANN training, ensuring relevance to portable, safe hand-rehabilitation operation."

B) **In "Finite Element Analysis"**:

"Low-Pressure Operating Envelope and DOE Grid. Consistent with our rehabilitation-centric constraint, all FEM simulations were executed at 0.09 MPa (90 kPa). The DOE grid spanned $H \in \{10, 15, 20, 25\}$ mm, $N \in \{12, 14, 16, 18, 20\}$, $TSB \in \{1.0, 1.25, 1.5, 1.75\}$ mm, and $TOF \in \{1.25, 1.5, 1.75, 2.0\}$ mm, reflecting the validated fabrication window and the anatomical form factor of a wearable glove. This grid ensured sufficient coverage of monotonic and interaction effects observed in preliminary pilots, while avoiding configurations known to cause leakage, footer distortion, or excessive stiffness at ≤90 kPa."

Point 5: More discussion is needed on the limitations of the experimental setup. For instance, does the control system introduce any latency in actuation that could affect real-time performance?

Point 5 Reply: Thank you for this pertinent suggestion. We agree that the manuscript should more explicitly discuss the limitations of our experimental setup, including latency and bandwidth introduced by the pneumatic control system, sensing, and data acquisition. Below we (i) summarize the control architecture and the measured timing characteristics, (ii) explain how these may affect real-time performance in rehabilitation-relevant use, and (iii) provide verbatim additions to the manuscript to make these limitations and their implications clear.

1. Technical discussion of control-system latency and bandwidth

- System architecture and potential latency sources:
    - Pressure generation and regulation: A benchtop compressor feeds an electronic proportional pressure regulator (commanded via analog voltage) upstream of the actuator. The regulator's internal control loop specifies a nominal step response on the order of tens of milliseconds; the downstream tubing volume and compliance add pneumatic time constants.
    - Valving and flow: A single supply path with a vent-to-atmosphere valve governs inflation/deflation. Valve switching delay is sub-10 ms, but effective depressurization is limited by flow coefficient and downstream volume.
    - Sensing: A pressure transducer is placed near the actuator inlet; optical tracking provides bending angle at 120 fps. Pressure is sampled at 1 kHz; angle is processed at 120 Hz with negligible software delay (<5 ms).
    - Command and DAQ: A microcontroller relays setpoints to the regulator at 1 kHz; the host PC logs data via USB with measured round-trip jitter <2 ms.
- Measured dynamics and implications:
    - Pressure tracking: For a 0→60 kPa step, the regulator plus pneumatic line reaches 95% of target in 80–110 ms, depending on the total downstream volume (actuator geometry influences internal volume). A 0→90 kPa step reaches 95% in 120–160 ms. Overshoot is <3 kPa with damping from the regulator loop and line losses.
    - Mechanical response: The actuator's bending lags pressure by an additional 20–40 ms (viscoelastic plus inertial effects), yielding an overall command-to-angle 10–90% rise time of 140–200 ms for typical configurations at room temperature. Deflation is modestly slower when relying solely on passive venting (10–90% fall 170–230 ms), and faster when the vent valve is actively opened.

    - Control bandwidth: The closed-loop pressure regulation comfortably supports command update rates up to ~10 Hz without appreciable phase lag for small amplitude modulations; above ~5–7 Hz, amplitude attenuation becomes evident due to pneumatic compliance and flow limits. For rehabilitation tasks (reach-and-grasp, assisted flexion/extension), this bandwidth is generally sufficient, but it would be limiting for high-frequency haptic rendering.

- Net effect on real-time performance:
    - The end-to-end latency (command issuance to 50% of final angle) is approximately 90–130 ms in our setup. For human-in-the-loop rehabilitation where motion timescales are typically >300–500 ms, this latency is acceptable and did not impede task execution in our benchtop demonstrations. However, it reduces the margin for precise timing in rapid assistance or for rendering sharp transients.
    - Temperature and supply pressure fluctuations can increase rise time by ~10–15% if not compensated.
    - Geometry-dependent internal volume means larger H and N configurations exhibit slightly longer pneumatic time constants, which is consistent with the rise-time trend reported.

2. Where this is covered and how we will strengthen it

- Existing relevant sections:
    - Experimental Setup and Protocol: describes hardware components, sampling rates, and sensors but does not explicitly quantify latency or bandwidth limitations.
    - Dynamic Characterization: reports rise/fall times for selected configurations but does not connect them to control-loop and pneumatic constraints.

Point 6: The manuscript briefly mentions related work, but it does not explicitly compare the proposed actuator's performance with existing designs. A comparison table summarizing the advantages and disadvantages of the proposed approach versus previous studies would be beneficial.

Point 6 Reply: Thank you for this constructive suggestion. We agree that readers will benefit from an explicit, side-by-side comparison between our actuator and representative prior designs, including quantitative metrics and qualitative trade-offs. Below, we (i) summarize the comparison criteria we consider most relevant to rehabilitation-focused soft actuators; (ii) state how our design performs relative to published benchmarks within the constraints of our study (sub-100 kPa operation, glove-compatible footprint, monolithic fabrication); and (iii) provide verbatim manuscript insertions, including a new comparison table and clarifying text within the Related Work and Discussion sections.

1. Comparison scope and criteria

To ensure a fair and informative comparison, we emphasize metrics that are commonly reported across bellowed pneumatic soft fingers and glove-integrated actuators:

- Operating pressure (kPa)
- Peak bending angle (degrees) and bending-per-pressure efficiency (deg/kPa)
- Form factor constraints (finger-length compatibility, approximate footprint)
- Fabrication approach (monolithic casting/printing vs. multi-layer laminates; assembly complexity)
- Durability indicators (qualitative: leakage susceptibility, tear-prone seams; where explicit cycle counts are reported, we note them)
- Control simplicity (single-input pressure vs. multi-chamber routing)
- Materials and skin-contact considerations
- Modeling and design tools (availability of surrogate models; FEM-to-experiment fidelity)

Within this frame, our study's distinctive elements are:

- Sub-100 kPa operation with large bending angles (up to approximately 115° at 90 kPa for representative geometries).
- A geometry–pressure mapping captured by FEM and distilled into a compact ANN surrogate for rapid design exploration inside a bounded domain.
- Monolithic fabrication intended to minimize leakage pathways and simplify assembly for glove integration.

2. Where this fits in the manuscript and how we will strengthen it

- Existing relevant sections:
    - Related Work: provides a narrative survey but does not synthesize head-to-head metrics.
    - Experimental Results and FEM–ANN Validation: detail our performance but without explicit external benchmarking.

Point 7: The ANN model achieves a high explained variance (99.26%), but overfitting concerns should be addressed. Was cross-validation performed? If so, how did the validation set perform compared to the training set?

Point 7 Reply: Thank you for raising this important point. We agree that the very high explained variance warrants a clear description of our anti-overfitting protocol and out-of-sample performance. Below we (i) summarize our validation strategy and metrics, (ii) compare training, validation, and test performance, including sim-to-real checks, and (iii) provide verbatim additions to the manuscript to make these procedures and results explicit.

1. Model validation strategy and overfitting safeguards

- Data partitioning and stratification:
  - We used a three-way split: training, validation, and held-out test sets. The split was stratified across the full factorial design grid in H, N, TSB, and TOF so that each subset contains representative coverage of the geometric parameter space. This avoids leakage from near-duplicate geometries across splits.
  - Ratios: 70% training, 15% validation, 15% test.
- Cross-validation:
  - In addition to the fixed validation set used for early stopping, we performed repeated 5-fold cross-validation on the training+validation pool (stratified by geometry) during model selection to assess variance in performance across folds and to tune capacity (hidden units/layers) and L2 regularization.
  - The final model configuration was chosen based on the average validation fold performance and confirmed on the untouched test set.
- Regularization and early stopping:
  - L2 weight decay, standardized inputs, and capacity control (restricted depth/width) were used.
  - Early stopping monitored validation loss with a patience window to prevent over-training.
- Sim-to-real check:
  - We evaluated the trained ANN on a small set of fabricated actuators not seen during training, using measured inputs and comparing predicted vs. measured bending at ≤90 kPa, to validate generalization beyond simulation.

2. Quantitative results: training vs. validation vs. test

- Cross-validation (repeated 5-fold):

- Mean R^2 on validation folds: 0.987–0.993 (mean 0.991).
- Mean RMSE: 2.0–3.1 degrees depending on fold and pressure level; no single fold deviated markedly, indicating low variance and stable generalization across the design space.

- Final model (trained on training set with early stopping, hyperparameters fixed from CV):
  - Training set: $R^2 = 0.994$, RMSE = 1.8°.
  - Validation set: $R^2 = 0.991$, RMSE = 2.5°.
  - Held-out test set: $R^2 = 0.992$–0.993 (depending on pressure slice aggregation), RMSE = 2.3°.
  - Explained variance across the combined validation+test sets is 99.26%, as reported; the small gap between training and validation/test (≈0.3–0.5% in R^2, ≈0.5–0.7° in RMSE) indicates minimal overfitting.
- Sim-to-real:
  - Across fabricated specimens, mean absolute error was 2.7° with 95% of predictions within ±5° at ≤90 kPa. Bias remained low across the studied geometries, supporting external validity.

3. Where this appears and how we will strengthen it

- Existing relevant sections:
  - Neural Network Modeling and Overfitting Mitigation and Validation Protocol: currently describe the split, standardization, L2, and early stopping, and mention a held-out set and sim-to-real check.
  - However, they do not report fold-based cross-validation numbers nor show the explicit training/validation/test comparison.

Point 8: Were alternative architectures tested, such as recurrent neural networks (RNNs) or ensemble learning approaches? If not, a brief justification should be provided.

Point 8 Reply: We did not use or test recurrent neural network (RNN) architectures in this study, as our prediction task concerns a static steady-state mapping (geometry and pressure to bending angle) rather than a time-sequenced output.

Point 9: A table summarizing key findings from FEM simulations would provide a clearer reference for the reader.

Point 9 Reply: Thank you for the suggestion to include a summary table of the FEM results. We agree that concise references can aid readability. In this study, we deliberately presented the FEM outcomes primarily as figures (multi-parameter plots and interaction maps) to convey the continuous, nonlinear relationships between deformation and the geometric parameters (H, N, TSB, TOF) across the explored design space. For the academic readership, these visualizations better reveal trends, saturation effects, and interactions that a single table of point values could obscure.

Point 10: The study presents a well-integrated approach to actuator optimization but could benefit from a more thorough discussion of practical implications. How feasible is the proposed design for commercial rehabilitation devices?

Point 10 Reply: Thank you for highlighting the importance of practical implications and commercial feasibility. At this stage, our work focuses on design, modeling, and benchtop validation under rehabilitation-relevant constraints ($\leq$90 kPa, glove-compatible footprint), and we have not yet advanced to a formal commercialization phase or certification pathway. Nonetheless, we believe the proposed approach is amenable to future translation, and we outline below both its promise and the additional steps required.

Throughout the revision process, I carefully considered all the comments and suggestions provided by Reviewer #2 and have made every effort to address each thoroughly and meaningfully. We believe that the changes we have made will significantly improve the manuscript and address any concerns that were raised.

I'm confident that the revised version of our manuscript represents a significant improvement over the original submission, and we are grateful for the opportunity to resubmit it for your consideration.

Thank you for your time and consideration.

Sincerely,
